# Early risk assessment model for sepsis-associated acute kidney injury combining quantitative contrast-enhanced ultrasound and biomarkers: a prospective pilot study

**DOI:** 10.3389/fmed.2026.1991344

**Published:** 2026-10-28

**Authors:** Yewen Zhan, Can Tang, Hong Pu

**Affiliations:** 1 Department of Critical Care Medicine, West China School of Medicine, West China Hospital of Sichuan University, Chengdu, China; 2 Department of Critical Care Medicine, West China Second School of Medicine, Sichuan University, Chengdu, China; 3 Department of Ultrasound Medicine, West China School of Medicine, West China Hospital of Sichuan University, Chengdu, China

**Keywords:** contrast-enhanced ultrasound, prediction model, renal microcirculation, ridge regression, sepsis-associated acute kidney injury

## Abstract

**Background:**

Sepsis-associated acute kidney injury (SA-AKI) is associated with high mortality in the intensive care unit (ICU), yet early diagnosis remains challenging due to the diagnostic lag of conventional functional biomarkers. We aimed to develop and validate a Ridge-penalized multimodal model integrating quantitative renal contrast-enhanced ultrasound (CEUS) with clinical biomarkers for early SA-AKI risk stratification.

**Methods:**

Adult septic patients were prospectively enrolled in a 35-bed general ICU. Quantitative renal CEUS parameters—including peak intensity (PI), time to peak (TTP), mean transit time (MTT), and area under the time-intensity curve (TIC-AUC)—and routine clinical variables were collected within 24 h of admission. SA-AKI was defined according to the Kidney Disease: Improving Global Outcomes criteria within 7 days. A multimodal Ridge logistic regression model (Model C) was developed using stratified 5-fold cross-validation (*λ*1SE rule) and compared with CEUS-only (Model A) and clinical-only (Model B) candidate models. Internal validation was performed using 1,000 bootstrap resamples. Multivariable Firth-penalized regression was used to evaluate independent predictors.

**Results:**

Among 63 analyzed patients, 25 (39.7%) developed SA-AKI at a median lead time of ICU day 2 (IQR: day 1–day 3) post-admission; none met AKI criteria at baseline. Quantitative CEUS revealed significant cortical hypoperfusion (PI and TIC-AUC) and prolonged microvascular transit (MTT) in the SA-AKI group (all *P* < 0.05), while other temporal or slope parameters showed non-significant trends. Model C achieved superior discrimination with a bias-corrected area under the curve (AUC) of 0.852 (95% CI: 0.789–0.932), outperforming Model A (0.818) and Model B (0.740). At the optimal threshold, Model C yielded 80.0% sensitivity and 84.2% specificity. Decision curve analysis confirmed higher standardized net benefit for Model C across threshold probabilities of 0.15–0.60. Adjusted age and baseline serum cystatin C confirmed lower PI (aOR = 0.31), lower TIC-AUC (aOR = 0.31), and prolonged TTP (aOR = 2.1) as independent predictors (all *P* < 0.05).

**Conclusions:**

Quantitative renal CEUS parameters are independent early indicators of SA-AKI. Integrating renal CEUS with clinical biomarkers offers a promising, noninvasive strategy for early SA-AKI risk stratification up to 48 h before clinical diagnosis.

**Clinical Trial Registration:**

https://www.chictr.org.cn/, identifier ChiCTR2300067267.

## 1 Introduction

Sepsis is a major driver of acute kidney injury (AKI) in the intensive care unit (ICU), and sepsis-associated AKI (SA-AKI) is linked to higher mortality, longer hospitalization, and increased costs (1–3). Because specific therapies remain limited, early risk stratification is clinically crucial (4, 5).

SA-AKI is clinically defined as the development of AKI in the presence of sepsis (6). However, standard AKI diagnosis is currently based on serum creatinine (Scr) and urine output (7), both of which lack sensitivity for early detection (4, 5). Although biomarkers such as serum cystatin C (sCys C) may enable earlier identification, their availability is still limited in many settings (8–10).

Contrast-enhanced ultrasound (CEUS) is a bedside imaging technique utilizing non-nephrotoxic intravascular microbubbles (11–15). Since microcirculatory dysfunction is central to SA-AKI pathogenesis (16–18), CEUS-derived time–intensity curve (TIC) metrics, such as peak intensity (PI), time to peak (TTP), mean transit time (MTT), and area under the curve (TIC-AUC), can capture early cortical perfusion abnormalities and may change before Scr rises (19–30). Although conventional Doppler-derived parameters, such as the renal resistive index (RRI), are widely used for bedside hemodynamic assessment, they primarily reflect macrovascular compliance and systemic factors rather than tissue-level renal microvascular perfusion (31–33).

Most existing SA-AKI prediction models rely on conventional clinical indices (34) and commonly use standard logistic regression, which can be unstable in small-sample, low events-per-variable (EPV) settings and when predictors are collinear (35, 36). Unlike Lasso, ridge regression performs coefficient shrinkage without dropping predictors, preserving the full spectrum of microvascular and clinical covariates (37, 38).

Therefore, following TRIPOD (Transparent Reporting of a multivariable prediction model for Individual Prognosis or Diagnosis) guidance (39), we developed and internally validated a ridge-based model that integrates quantitative renal CEUS parameters with routine clinical indicators to improve early prediction of SA-AKI.

## 2 Materials and methods

### 2.1 Study design and participants

This prospective exploratory cohort study, conducted at West China Shangjin Hospital, Sichuan University, from June 2023 to December 2025, consecutively screened ICU adult patients who met the Sepsis 3.0 International Consensus Criteria (40). Exclusion criteria were: (1) fulfillment of Kidney Disease: Improving Global Outcomes (KDIGO) criteria for AKI (at any stage, Stage 1–3) upon or within the first 24 h of ICU admission; (2) unavailable baseline renal function or suboptimal ultrasound images; (3) contraindications to sulphur hexafluoride microbubbles (e.g., allergy, recent acute coronary syndrome, severe arrhythmia, right-to-left cardiac shunt, severe pulmonary hypertension, or adult respiratory distress syndrome); (4) history of kidney transplant or severe urinary system diseases; (5) pregnancy or breastfeeding; (6) severe cardiopulmonary insufficiency with an estimated life expectancy under 72 h; and (7) treatment with highly nephrotoxic drugs. In accordance with the 2020 KDIGO Consensus (41), SA-AKI was defined as the development of AKI[according to the KDIGO criteria (7)] within 7 days of ICU admission among sepsis patients. Patients were divided into non-SA-AKI and SA-AKI groups based on whether SA-AKI occurred within 7 days.

The Ethics Committee of West China Hospital of Sichuan University has reviewed and approved this research project (Approval No. 2022-1374). All subjects or their authorised agents have given informed consent and signed the informed consent form.

### 2.2 Clinical data collection and predictor variables

Within 24 h of ICU admission, demographic, clinical, and laboratory data were collected using standardised methods. All data collectors were appropriately trained to ensure consistency and accuracy. Demographic variables included age, gender, body mass index (BMI), comorbidities, medication history, surgical history, and sources of infection. Disease severity was assessed using the Acute Physiology and Chronic Health Evaluation II (APACHE II) score and the Sequential Organ Failure Assessment (SOFA) score. Clinical variables included mean arterial pressure (MAP), central venous pressure (CVP), and use of vasoactive drugs. Laboratory variables included Scr, sCys C, estimated glomerular filtration rate (eGFR), B-type natriuretic peptide (BNP), procalcitonin (PCT), interleukin-6 (IL-6), and C-reactive protein (CRP).

### 2.3 Ultrasonography and CEUS assessment

Both groups underwent CEUS on Admission Day 1 under a double-blind, interdisciplinary quality-control framework. Image acquisition and TIC extraction were conducted per standardised protocol by a dedicated team (a radiologist with >10 years' experience, assisted during the procedure by an ICU physician independent of direct patient care), both blinded to baseline clinical data, group assignment, and outcomes. Final image loops and quantitative results were audited by the senior radiologist, requiring standardised planes, clear microbubble perfusion, and accurate parameter measurements for study inclusion.

Examinations used a Mindray M9 scanner with a C5-1 transducer and SonoVue (Bracco International B.V., Milan, Italy). Settings included: centre frequency 2.2 MHz, dynamic range 110 dB, acoustic power ∼4.8%, and mechanical index 0.06–0.08 to prevent bubble destruction. Contrast gain was tuned to suppress background tissue, and speckle reduction was enabled to maximise the signal-to-noise ratio.

#### 2.3.1 CEUS examination protocol

Patients were positioned in lateral decubitus to obtain the maximal longitudinal coronal plane for each kidney. SonoVue (0.02 mL/kg) was administered via an antecubital bolus followed by a 5 mL saline flush, with timing initiated simultaneously while holding the transducer stationary. Continuous 3-minute cine loops were saved. A 15-minute washout period was allowed before evaluating the contralateral kidney using the same protocol. **TIC Analysis:** Image analysis was performed using Mindray M9 quantification software. Three 5 mm cortical ROIs were placed at a consistent depth per kidney, excluding the medulla and large intrarenal vessels. Raw TICs were fitted via a lognormal distribution model to derive cortical perfusion parameters (Figure 1): TTP, PI, MTT, TIC-AUC, arrival time (AT), ascent slope (AS), descent slope.

**Figure 1:**
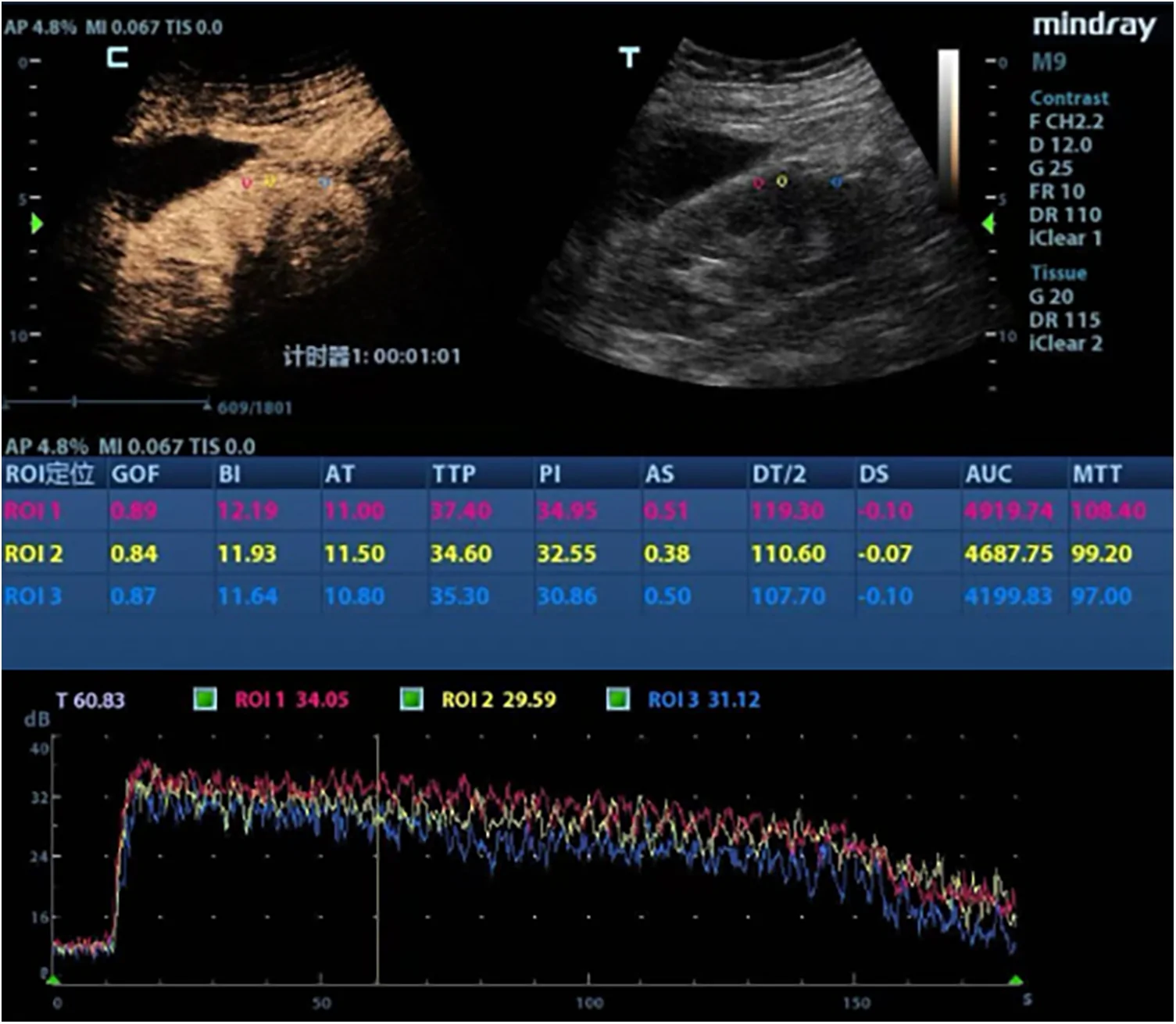
Quantitative renal contrast-enhanced ultrasound (CEUS) imaging and time–intensity curve (TIC) analysis. (Top) Dual-display mode showing the CEUS perfusion image (left, C) and matching grayscale B-mode image (right, T) of the renal cortex. Three interest regions (ROI 1, ROI 2, and ROI 3) are designated within the renal cortex for quantitative perfusion evaluation. (Middle) Quantitative microvascular perfusion parameters automatically extracted from the software, including arrival time (AT), time to peak (TTP), peak intensity (PI), ascent slope (AS), area under the curve (AUC), and mean transit time (MTT). (Bottom) Corresponding time–intensity curves (TIC) derived from the three cortical ROIs showing the enhancement kinetics over time. AS, ascent slope; AT, arrival time; AUC, area under the curve; BI, base intensity; CEUS, contrast-enhanced ultrasound; DS, descent slope; DT/2, half-time of wash-out (Descent Half-Time); GOF, goodness of fit; MTT, mean transit time; PI, peak intensity; ROI, region of interest; TIC, time–intensity curve; TTP, time to peak.

For final quantitative analysis, the right kidney was uniformly selected over the left to maximise image quality and TIC curve-fitting reliability. Unlike the right kidney which benefits from the liver as an excellent acoustic window, the left kidney lacks such an advantage and was frequently compromised by bowel gas in our critically ill patients (42, 43). Each parameter represented the average of three replicates measured at a uniform depth in the right renal cortex.

### 2.4 Outcome definition

All enrolled patients were prospectively followed up for up to 28 days post-ICU admission (or until discharge/death), with clinical and outcome data retrieved from the electronic medical record system. The primary outcome was the incidence of SA-AKI occurring within 7 days of ICU admission. The secondary outcomes included: (1) SA-AKI recovery or reversal status within 14 days; (2) 28-day all-cause mortality; (3) Length of ICU stay (in days); (4) Duration of mechanical ventilation (MV, in hours); and (5) Total hospitalisation costs.

### 2.5 Statistical analysis and model development

All statistical analyses were performed using R software (version 4.5.1).

#### 2.5.1 Descriptive statistics

Data normality was assessed using the Shapiro–Wilk test. Continuous variables are presented as median [interquartile range, (Q1, Q3)] and compared using the Mann–Whitney *U*-test. Categorical variables are presented as *n*(%) and compared using the Pearson chi-square test or Fisher's exact test, as appropriate.

#### 2.5.2 Data preprocessing

Raw data underwent rigorous quality control prior to model construction. Laboratory parameters with threshold limits (e.g., “<” or “>”) were standardized to numerical values. Skewed continuous variables (BNP, PCT, and IL-6) were log-transformed (log1p) to normalize distributions and reduce extreme-value influence.

To maximize statistical power and minimize selection bias in this limited cohort, missing data were systematically assessed and imputed. Fisher's exact test was initially applied to assess the association between variable missingness and SA-AKI. Anthropometric variables (height, weight, and BMI) were frequently missing because critical illness, deep sedation, or MV precluded standing measurements. Because key determinants of missingness (e.g., MV status, APACHE II, and SOFA scores) were fully recorded, missing data were assumed to be Missing at Random (MAR) conditional on these observed variables.

Under the MAR assumption, missing values were imputed using Multivariate Imputation by Chained Equations (MICE, via the mice package in R). Predictive Mean Matching (PMM) was used for continuous variables and logistic regression for categorical variables. The imputation model incorporated all predictor variables and clinical indicators of disease severity. The outcome variable (SA-AKI occurrence) was included as a predictor to inform imputation but was not itself imputed. Five imputed datasets (m = 5) were generated, and statistical estimates were pooled using Rubin's rules.

#### 2.5.3 Model development

Model development and reporting followed the TRIPOD (39) statement. Given the exploratory design and limited sample size, candidate predictors were not excluded solely to meet conventional EPV rules. From a pathophysiological perspective, quantitative CEUS parameters—such as PI (reflecting microvascular blood volume) and TTP (reflecting perfusion velocity)—are inherently correlated and jointly characterize renal microvascular hemodynamics.

To address multicollinearity and reduce overfitting, we used ridge (L2-penalized) logistic regression. The optimal regularization parameter (*λ*) was selected via stratified 5-fold cross-validation using the conservative 1-SE rule (*λ*_1SE_, defined as the largest penalty within one standard error of the minimum binomial deviance). This approach shrinks coefficients toward zero, constraining model complexity and improving internal generalizability while retaining the full set of clinically relevant predictors.

To prevent data leakage, all components used to diagnose AKI per KDIGO criteria—including Scr, urine output, and eGFR—were excluded from model development.

To evaluate the incremental predictive value of CEUS metrics, we constructed three models representing different clinical dimensions using *a priori* selected predictors:

Model A (CEUS-only Model): Constructed as the baseline ultrasound model based on established physiological rationale (19–30), incorporating four key continuous CEUS parameters (MTT, PI, TIC-AUC, and TTP) to evaluate intrinsic imaging-derived predictive performance.

Model B (Clinical-only Model): Designed to reflect routine non-ultrasound clinical and biochemical assessment. Selecting predictors across distinct pathophysiological domains—baseline physiological reserve, systemic inflammation, volume status, and renal function—this model incorporated age, PCT, BNP, and sCys C.

Model C (Combined Multimodal Model): Serving as the primary predictive tool, this comprehensive model integrated both quantitative CEUS parameters and routine clinical features to maximize early diagnostic capability for SA-AKI. Model C was parameterized into a continuous mathematical equation to yield individualized predicted probabilities of SA-AKI for clinical application.

#### 2.5.4 Model validation

Given the limited sample size, which precluded an independent external validation cohort, internal validation and performance assessment were performed using bootstrap resampling (B = 1000; rms package in R).

##### 2.5.4.1 Discrimination

Discrimination was assessed using the apparent area under the curve (AUC). Model optimism was estimated via bootstrap resampling to obtain the bias-corrected AUC with a 95% confidence interval (CI). Using the apparent dataset, the optimal cutoff was identified by the Youden index, and sensitivity, specificity, negative predictive value (NPV), and positive predictive value (PPV) were calculated.

##### 2.5.4.2 Overall prediction error

The bias-corrected Brier score was computed to quantify overall prediction error and the absolute difference between predicted probabilities and observed outcomes.

##### 2.5.4.3 Calibration

Linear predictors (log-odds) were extracted for each bootstrap sample. To avoid non-convergence of maximum likelihood estimation due to separation in small samples, Firth's penalised likelihood logistic regression was then fitted with the linear predictor as the sole covariate. The calibration slope (ideal value: 1.0) and calibration intercept (ideal value: 0) were reported, along with non-parametrically smoothed calibration curves.

##### 2.5.4.4 Clinical utility

Decision curve analysis (DCA; dcurve package in R) was used to quantify net benefit across a range of threshold probabilities. Bootstrap-based optimism correction was applied to the DCA curves to provide an objective assessment of clinical utility.

#### 2.5.5 Sensitivity analyses

To evaluate the robustness of the combined model and main conclusions, multi-dimensional sensitivity analyses were performed. Fluctuations in regression coefficients and discrimination were reassessed by varying missing value imputation strategies, replacing key predictors, and forcibly including potential clinical confounders. (As the selection of primary study variables was grounded in clinical rationale and available literature, parameters for these sensitivity models were incorporated *post hoc* based on subsequent analytical findings). The specific sensitivity models were structured as follows: Model S1 (MICE m = 10): Imputation iterations were increased from 5 to 10 (m = 10) to test model stability against imputation variability. Model S2 (IL-6 Substitution): IL-6 replaced PCT to evaluate the impact of alternative inflammatory biomarkers on model performance. Model S3 (BMI Inclusion): BMI (high missingness) was forcibly included to examine whether imputing high-missingness variables alters the independent predictive value of CEUS metrics. Model S4 (Sex Adjustment): Sex was forcibly added as a covariate to the primary model. Model S5 (Comorbidity Adjustment): History of diabetes and abdominal surgery were forcibly added as potential clinical confounders. Model S6 (Comprehensive CEUS Panel): All available quantitative renal CEUS parameters were forcibly incorporated to evaluate whether potential collinearity among microvascular parameters compromised model stability.

#### 2.5.6 Exploratory association and model interpretability analysis

To identify clinical and CEUS predictors associated with SA-AKI and enhance the interpretability of our predictive models, logistic regression analyses were performed using the multiple imputed datasets:

##### 2.5.6.1 Univariable and multivariable firth logistic regression

Univariable logistic regression was first applied to estimate crude odds ratios (ORs) and 95%CI for candidate predictors using Rubin's rules. To mitigate potential data separation and parameter-estimation bias induced by small sample sizes and low EPV, multivariable analysis was conducted using Firth's penalized likelihood logistic regression (logistf package in R), which incorporates a Jeffreys prior penalty to yield robust, unbiased coefficient estimates.

##### 2.5.6.2 Standardization of continuous predictors

To eliminate dimensional variation across parameters with differing physical units and enable direct comparison of effect sizes, all non-binary continuous predictors (including CEUS parameters and clinical metrics) were standardized into Z-scores. Their associations with SA-AKI risk are reported per 1-standard deviation (1-SD) increment.

##### 2.5.6.3 Independent evaluation of CEUS parameters

When evaluating individual CEUS parameters, both unadjusted and multivariable-adjusted models—controlling for age and baseline sCys C—were fitted to determine their independent contribution to SA-AKI risk.

## 3 Results

### 3.1 Baseline characteristics and clinical outcomes

Initially, 81 septic patients were screened, with zero contrast-related adverse events (0%). 18 patients were excluded (severe cardiopulmonary insufficiency [*n* = 3], chronic kidney disease [*n* = 6], refusal [*n* = 4], and hemodynamic instability preventing CEUS [*n* = 5]).

Ultimately, 63 patients were analyzed [52 males [82.5%]; median age, 60.0 years [IQR: 50.0–72.0]]. Patients were stratified into non–SA-AKI (*n* = 38, 60.3%) and SA-AKI groups (*n* = 25, 39.7%). Baseline characteristics—including age, sex, BMI, APACHE II, SOFA, MAP, admission source, and primary infection site—were comparable (all *P* > 0.05). CVP differed statistically (*P* = 0.024) but shared an identical median [ 6.0 (5.0, 6.0) vs. 6.0 (6.0, 7.0) cmH₂O], offering minimal clinical significance. Diabetes mellitus prevalence was higher in the SA-AKI group (36% vs. 13%, *P* = 0.033). Main baseline demographic and key clinical metrics are summarized in Table 1, with detailed extended variables (including specific infection sites and lifestyle factors) provided in Supplementary Table S1.

**Table 1:** Baseline characteristics and clinical outcomes of the study population.

Characteristic	Overall (*N* = 63)	Non-SA-AKI (*N* = 38)	SA-AKI (*N* = 25)	*P* Value
**Demographics & Clinical Scores**				
Age, years	60.0 (50.0, 72.0)	55.0 (52.0, 70.0)	71.0 (48.0, 72.0)	0.257
Male sex, *n* (%)	52 (83%)	29 (76%)	23 (92%)	0.176
APACHE II score	20.0 (14.0, 28.0)	20.0 (13.0, 28.0)	21.0 (14.0, 28.0)	0.994
SOFA score	7.0 (5.0, 9.0)	7.0 (5.0, 8.0)	9.0 (5.0, 12.0)	0.122
MAP, mmHg	86.0 (81.0, 96.0)	85.0 (79.0, 95.0)	88.0 (84.0, 96.0)	0.513
CVP, mmHg	6.0 (5.0, 7.0)	6.0 (5.0, 6.0)	6.0 (6.0, 7.0)	**0** **.** **024**
**Comorbidities & Source of Infection**				
Hypertension, *n* (%)	16 (25%)	8 (21%)	8 (32%)	0.329
Diabetes mellitus, *n* (%)	14 (22%)	5 (13%)	9 (36%)	**0** **.** **033**
Pulmonary site of infection, *n* (%)	43 (69%)	24 (63%)	19 (76%)	0.284
Vasoactive drug use, *n* (%)	20 (32%)	9 (24%)	11 (44%)	0.090
**Laboratory Biomarkers**				
Scr, µmol/L	67.0 (56.0, 82.0)	65.0 (46.0, 73.0)	72.0 (66.0, 92.0)	**0** **.** **018**
eGFR, mL/min/1.73 m^2^	86.5 (70.9, 99.5)	91.1 (78.0, 100.5)	72.7 (66.5, 88.9)	**0** **.** **010**
sCys C, mg/L	1.21 (1.06, 1.87)	1.14 (1.00, 1.47)	1.84 (1.20, 2.37)	**0** **.** **004**
BNP, pg/mL	2,104.5 (889.0, 6,541.0)	1,510.5 (323.5, 3,590.0)	5,706.5 (1,884.0, 8,119.0)	**0** **.** **002**
PCT, ng/mL	5.85 (0.88, 15.93)	2.45 (0.49, 12.78)	7.07 (3.78, 32.33)	**0** **.** **013**
**Clinical Outcomes**				
ICU length of stay, days	7.0 (4.0, 14.0)	6.0 (2.0, 9.0)	8.5 (5.0, 19.0)	**0** **.** **009**
28-day mortality, *n* (%)	10 (16%)	3 (7.9%)	7 (28%)	0.042
Invasive mechanical ventilation, *n* (%)	47 (75%)	28 (74%)	19 (76%)	0.836
Total hospitalization cost, RMB	101,456 (51,225, 167,919)	58,675 (43,103, 127,410)	114,138 (74,475, 276,392)	**0** **.** **002**
14-day renal outcomes, *n* (%)				**<0** **.** **001**
No AKI	48 (76%)	35 (92%)	13 (52%)	
Stage 1 AKI	6 (9.5%)	2 (5.3%)	4 (16%)	
Stage 2 AKI	2 (3.2%)	1 (2.6%)	1 (4.0%)	
Stage 3 AKI	7 (11%)	0 (0%)	7 (28%)	

AKI, acute kidney injury; APACHE II, Acute Physiology and Chronic Health Evaluation II; BNP, brain natriuretic peptide; CVP, central venous pressure; eGFR, estimated glomerular filtration rate; ICU, intensive care unit; MAP, mean arterial pressure; PCT, procalcitonin; SA-AKI, sepsis-associated acute kidney injury; sCR, serum creatinine; sCys C, serum cystatin C; SOFA, Sequential Organ Failure Assessment. Data are expressed as median [IQR] for continuous variables and count (%) for categorical variables. Comparisons were performed using the Mann–Whitney *U*-test, Chi-square test, or Fisher's exact test, as appropriate. Bold *P* values indicate statistical significance (*P* < 0.05).

Laboratory results showed comparable BUN (*P* = 0.762) and CRP (*P* = 0.142). However, sCys C (*P* = 0.004), Scr (*P* = 0.018), BNP (*P* = 0.002), and PCT (*P* = 0.013) were significantly higher, whereas eGFR (*P* = 0.010) was lower in the SA-AKI group. Notably, none of the 25 SA-AKI patients met the diagnostic criteria for AKI within the first 24 h of ICU admission (i.e., during the baseline CEUS assessment). To clarify the temporal sequence between microvascular alterations and clinical functional impairment, we conducted a retrospective analysis and found that the median time from ICU admission to fulfilling KDIGO diagnostic criteria in the SA-AKI group was approximately Day 2 (IQR: Day 1–Day 3).

Day-14 renal outcomes (KDIGO stage) differed significantly (*P* < 0.001). Most non–SA-AKI patients (92.1%) remained AKI-free, whereas 28.0% of SA-AKI patients reached/maintained Stage 3 AKI; notably, 52.0% recovered (no AKI). Furthermore, SA-AKI was associated with higher 28-day mortality (*P* = 0.042), longer ICU stay (8.5 vs. 6 days, *P* = 0.009), and increased total hospital costs (114,138 vs.58,675, *P* = 0.002).

### 3.2 Quantitative renal CEUS parameters

Quantitative CEUS parameters differed significantly between groups (Table 2). Patients with SA-AKI exhibited a renal cortical perfusion pattern of hypoperfusion and reduced flow velocity: significantly lower PI [29.80 (28.70–32.20) vs. 34.00 (32.00–36.41) dB, *P* < 0.001] and TIC-AUC [3,246.30 (2,868.80–3,725.90) vs. 4,179.30 (3,591.60–4,695.90) a.u., *P* < 0.001], alongside prolonged MTT [97.60 (80.40–106.30) vs. 81.82 (71.20–93.20) s, *P* = 0.013]. Trends toward prolonged AT (*P* = 0.062) and decreased AS (*P* = 0.073) were also observed in the SA-AKI group.

**Table 2:** Quantitative renal CEUS parameters in patients With vs. Without SA-AKI.

Characteristic	Overall (*N* = 63)	Non-SA-AKI (*N* = 38)	SA-AKI (*N* = 25)	*P* Value
AT, s	13.90 (8.10, 17.20)	13.35 (6.90, 16.20)	14.80 (11.10, 21.60)	0.062
TTP, s	37.70 (29.40, 43.70)	32.80 (28.50, 43.70)	39.10 (29.80, 45.30)	0.156
MTT, s	87.60 (73.90, 97.70)	81.82 (71.20, 93.20)	97.60 (80.40, 106.30)	**0** **.** **013**
PI, dB	32.50 (29.80, 35.50)	34.00 (32.00, 36.41)	29.80 (28.70, 32.20)	**<0**.**001**
TIC-AUC, a.u.	3,916 (3,108, 4,449)	4,179 (3,591, 4,695)	3,246 (2,868, 3,725)	**<0**.**001**
AS, dB/s	0.41 (0.37, 0.53)	0.42 (0.38, 0.60)	0.39 (0.30, 0.50)	0.073

AKI, acute kidney injury; AS, ascending slope; AT, arrival time; CEUS, contrast-enhanced ultrasound; DS, descending slope; MTT, mean transit time; PI, peak intensity; SA-AKI, sepsis-associated acute kidney injury; TIC-AUC, area under the time-intensity curve; TTP, time to peak.

Continuous CEUS parameters are expressed as median [IQR] and were compared between groups using the Mann–Whitney *U*-test. CEUS quantitative analysis was performed using time–intensity curve (TIC) software. Bold *P* values indicate statistical significance (*P* < 0.05).

### 3.3 Model development & specification

To evaluate the incremental predictive value of quantitative CEUS parameters beyond routine clinical biomarkers while managing potential multicollinearity, multivariable Ridge logistic regression was performed using stratified 5-fold cross-validation with the optimal penalty parameter (*λ*_1SE_).

The complete matrix of regularized coefficients across candidate models (Model A: CEUS-only; Model B: Clinical-only; Model C: Combined) is detailed in Supplementary Table S2). The explicit linear predictor formulation for the full multimodal model (Model C) is defined as:

$$\begin{array}{rl} \mathrm{logit}\left(P_{\mathrm{Model}\;C}\right)= & -0.8117+0.3415\cdot sCysC\\ & +0.1197\cdot BNP+0.1018\cdot PCT\\ & +0.0047\cdot \mathrm{Age}-0.0625\cdot PI\\ & +0.0151\cdot MTT+0.0058\cdot TTP\\ & -0.0003\cdot TIC-AUC \end{array}$$

The predicted individual probability of SA-AKI is derived via the sigmoid transformation:

$$P(\mathrm{SA}\text{-}\mathrm{AKI})=1/[1+\exp\;(-\mathrm{logit}(P_{\mathrm{Model}\;C})))]$$

### 3.4 Model performance

#### 3.4.1 Discrimination and classification performance

Following 1,000-iteration Bootstrap resampling, the combined model (Model C) achieved the highest discriminative capacity for predicting SA-AKI, with a bias-corrected AUC of 0.852 (95% CI: 0.789–0.932). Standalone quantitative CEUS parameters (Model A) demonstrated favorable performance, with a bias-corrected AUC of 0.818 (95% CI: 0.739–0.913). Both models significantly outperformed traditional clinical and renal biomarkers alone (Model B: bias-corrected AUC = 0.740, 95% CI: 0.637–0.837).

At the optimal probability threshold of 0.417 (determined by the Youden index), Model C provided the most balanced diagnostic performance, achieving a sensitivity of 80.0%, specificity of 84.2%, PPV of 76.9%, and NPV of 86.5%. Model A maintained a strong diagnostic yield at a threshold of 0.408 (sensitivity: 76.0%; specificity: 81.6%; PPV: 73.1%; NPV: 83.8%). Although Model B exhibited a higher sensitivity (88.0% at cutoff 0.372), its lower specificity (60.5%) and PPV (59.5%) resulted in a higher false-positive rate. (Table 3).

**Table 3:** Performance comparison of prediction models.

Model	AUC (95% CI)	Calibration Slope	Calibration Intercept	Brier Score	Optimal Cutoff	Sensitivity (%)	Specificity (%)	PPV (%)	NPV (%)
Model A (CEUS-only)	0.818 (0.739–0.913)	2.100	0.423	0.175	0.408	76.0	81.6	73.1	83.8
Model B (Clinical-only)	0.740 (0.637–0.837)	4.604	1.510	0.225	0.372	88.0	60.5	59.5	88.5
Model C (Combined)	0.852 (0.789–0.932)	3.148	0.815	0.194	0.417	80.0	84.2	76.9	86.5

AUC, area under the receiver operating characteristic curve; CEUS, contrast-enhanced ultrasound; CI, confidence interval; NPV, negative predictive value; PPV, positive predictive value.

AUC, calibration slope, calibration intercept, and Brier score represent optimism-corrected estimates generated via Bootstrap resampling (*B* = 1,000). A Brier score closer to 0 indicates superior prediction accuracy.

#### 3.4.2 Overall prediction accuracy and calibration

Regarding overall prediction accuracy, Model A and Model C outperformed Model B, maintaining lower apparent and bias-corrected Brier scores (Model A: 0.168/0.175; Model C: 0.168/0.194 vs. Model B: 0.222/0.225; Table 3). In calibration evaluations, Model A and Model C displayed calibration curves closer to the ideal trajectory (slope = 1, intercept = 0) than Model B, with calibration slopes (intercepts) of 2.100 (0.423) for Model A, 3.148 (0.815) for Model C, and 4.604 (1.510) for Model B (Figure 2A, Table 3).

**Figure 2:**
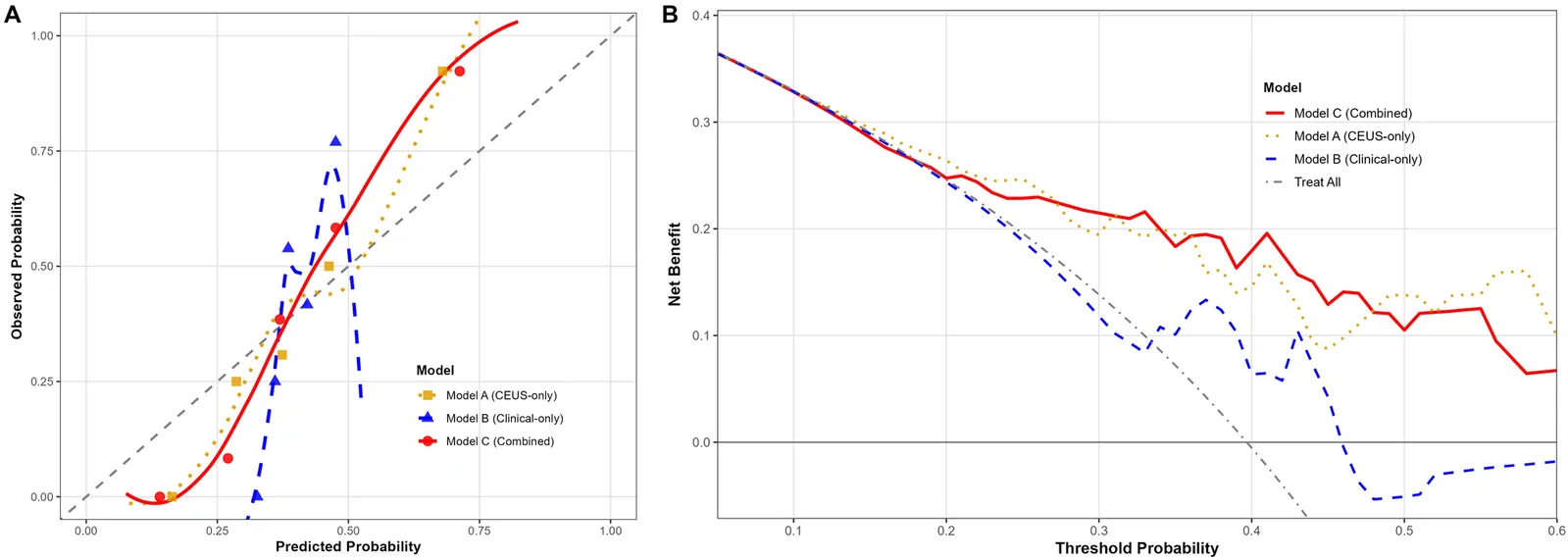
Calibration curves and decision curve analysis (DCA) comparing the predictive performance of candidate models. **(A)** Calibration curves of Model A (CEUS-only, yellow dotted line), Model B (Clinical-only, blue dashed line), and Model C (Combined, red solid line). The diagonal dashed grey line represents perfect calibration. **(B)** Decision curve analysis demonstrating the clinical net benefit across various threshold probabilities for Model A (yellow dotted line), Model B (blue dashed line), and Model C (red solid line). The grey solid line and grey short-dashed line represent the assumptions of treating all patients and treating no patients, respectively. CEUS, Contrast-Enhanced Ultrasound; SA-AKI, Sepsis-Associated Acute Kidney Injury.

#### 3.4.3 Clinical utility via decision curve analysis

Model C and Model A provided a higher standardized net benefit compared to Model B, as well as the “Treat All” and “Treat None” strategies across a wide threshold probability spectrum (0.15–0.60) (Figure 2B). In contrast, Model B's net benefit dropped rapidly, falling below the “Treat All” baseline at threshold probabilities exceeding 0.30 and dipping below zero beyond 0.45.

#### 3.4.4 Sensitivity analysi*s*

To confirm the robustness of Model C, sensitivity analyses were conducted across six prespecified scenarios (Supplementary Figure S2). Bias-corrected AUCs remained remarkably stable across all scenarios (0.842–0.854), aligning closely with the primary model (Model C, AUC = 0.852).

### 3.5 Exploratory analysis of independent predictor for SA-AKI

#### 3.5.1 Univariable logistic regression analysis

To assess preliminary associations between candidate predictors and SA-AKI, we performed univariable binary logistic regression on the multiply imputed datasets and pooled estimates using Rubin's rules (Figure 3). To facilitate comparability across predictors, all non-binary continuous variables were standardized and interpreted per 1-SD increase.

**Figure 3:**
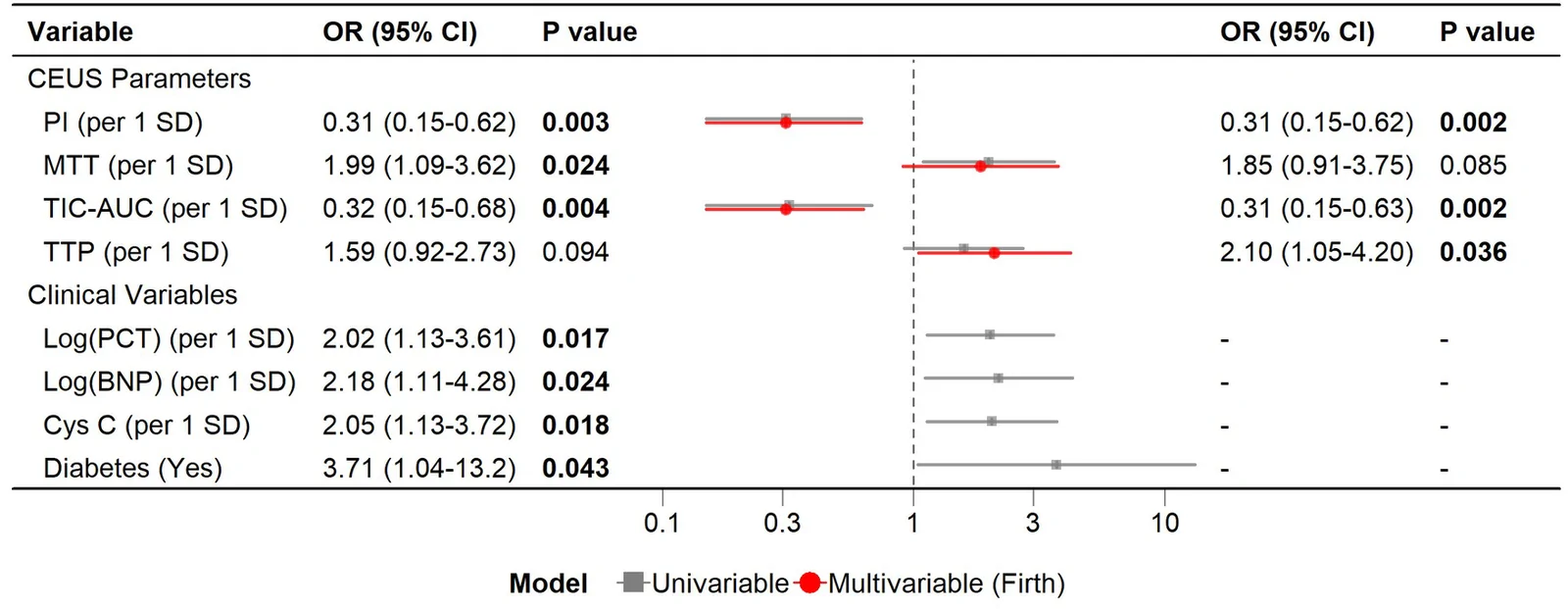
Forest plot of univariable and multivariable Firth penalized logistic regression analyses identifying predictors of sepsis-associated acute kidney injury (SA-AKI). The plot displays the odds ratios (OR) and 95% confidence intervals (CI) for quantitative renal contrast-enhanced ultrasound (CEUS) parameters and clinical variables. Grey squares represent univariable analysis results, while red circles represent multivariable Firth regression results (adjusted for age and baseline serum cystatin C). Bold P values indicate statistical significance (*P* < 0.05). Abbreviations: CEUS, contrast-enhanced ultrasound; PI, peak intensity; MTT, mean transit time; TIC-AUC, area under the time-intensity curve; TTP, time to peak; PCT, procalcitonin; BNP, B-type natriuretic peptide; Cys C, cystatin C; SD, standard deviation.

Among CEUS parameters, PI and TIC-AUC were significant protective factors; each 1-SD increase was associated with a substantial reduction in SA-AKI risk (PI: OR = 0.31, 95% CI: 0.15–0.62, *P* = 0.003; TIC-AUC: OR = 0.32, 95% CI: 0.15–0.68, *P* = 0.004). In contrast, prolonged MTT was associated with increased SA-AKI risk (OR = 1.99, 95% CI: 1.09–3.62, *P* = 0.024). TTP showed a nonsignificant trend toward increased risk (OR = 1.59, 95% CI: 0.92–2.73, *P* = 0.094).

Regarding laboratory indicators, standardized sCys C was significantly associated with higher SA-AKI risk (per 1-SD increase: OR = 2.05, 95% CI: 1.13–3.72, *P* = 0.018). Similarly, log-transformed PCT (OR = 2.02, 95% CI: 1.13–3.61, *P* = 0.017) and log-transformed BNP (OR = 2.18, 95% CI: 1.11–4.28, *P* = 0.024) were positively associated with SA-AKI risk. IL-6 was not significantly associated (*P* = 0.106).

Among clinical characteristics, history of diabetes mellitus was a significant risk factor for SA-AKI (OR = 3.71, 95% CI: 1.04–13.2, *P* = 0.043). Neither age (*P* = 0.691) nor BMI (*P* = 0.500) was significantly associated with the outcome.

#### 3.5.2 Independent predictive value of CEUS parameters

To assess whether CEUS parameters were independently associated with SA-AKI beyond age and baseline renal function, we performed multivariable Firth's penalized logistic regression (Figure 3). All models were adjusted for age and baseline sCys C, based on clinical relevance and univariable results. ORs for CEUS metrics are reported per 1-SD increase:

1. Independent associations of PI and TIC-AUC: PI and TIC-AUC remained significant protective factors after adjustment (PI: aOR = 0.31, 95% CI: 0.15–0.62, *P* = 0.002; TIC-AUC: aOR = 0.31, 95% CI: 0.15–0.63, *P* = 0.002), indicating that lower cortical perfusion intensity was independently associated with higher SA-AKI risk.
2. Changes in temporal-phase parameters after adjustment: Although TTP was not significant in univariable analysis, prolonged TTP emerged as an independent predictor after adjustment (aOR = 2.1, 95% CI: 1.05–4.20, *P* = 0.036). The association for MTT was attenuated and no longer statistically significant (aOR = 1.85, 95% CI: 0.91–3.75, *P* = 0.085), although the direction remained consistent.

Overall, multivariable Firth-adjusted regression confirmed that lower PI, reduced TIC-AUC, and prolonged TTP remained significantly associated with SA-AKI, independent of age and baseline renal function.

## 4 Discussion

In this prospective exploratory study, we introduced a Ridge-penalized logistic regression approach that integrates quantitative CEUS renal microvascular parameters with routine clinical biomarkers to construct an early predictive model for SA-AKI. Following optimism correction via 1,000 bootstrap iterations, the combined model demonstrated superior discriminative performance (bias-corrected AUC: 0.852), a lower overall calibration error (Brier score: 0.194), and broad clinical net benefit across threshold probabilities of 0.15–0.60, outperforming both the standalone clinical model (Model B, AUC: 0.740) and the standalone CEUS model (Model A, AUC: 0.818).

This study yields two principal clinical insights: 1. Quantitative CEUS accurately captures cortical microvascular dysfunction characterized by “low perfusion and delayed flow velocity” at ICU admission, establishing a median early warning window of 2 days (IQR: 1–3 days) prior to clinical diagnosis. 2. After controlling for age and baseline renal functional reserve (sCys C) using Firth penalized likelihood regression (to mitigate small-sample bias and separation), renal microvascular perfusion metrics—specifically PI, TIC-AUC, and TTP—remained strongly and independently associated with SA-AKI risk.

Regarding clinical outcomes, patients with SA-AKI exhibited higher 28-day mortality (28.0% vs. 7.9%), a greater proportion of Stage 3 AKI, longer ICU stays, and substantially increased total hospital costs, highlighting the heavy clinical and economic burden of SA-AKI (1–3, 44). Contrary to some prior reports (3), the rate of MV did not differ significantly between groups (*P* = 0.836). This is primarily attributable to the high baseline severity of our cohort—predominantly driven by severe pneumonia (68.3%)—where initial ventilatory demand was already exceptionally elevated (>70%). Consequently, the onset of AKI did not alter the absolute ventilation rate, but instead manifested as prolonged MV, extended ICU stay, and escalated healthcare costs. Notably, 52.0% of SA-AKI patients achieved renal recovery (no AKI) within 14 days, indicating that early microvascular impairment possesses inherent pathophysiological reversibility and underscoring a critical “golden window” for early recognition and intervention (4, 5).

Traditional KDIGO diagnostic criteria rely on Scr, a functional biomarker that rises laggingly only after more than 50% of functional nephrons are impaired (5), and one that is easily confounded by fluid resuscitation and muscle mass (40). Our timeline analysis showed that no SA-AKI patient met KDIGO diagnostic criteria within the first 24 h of ICU admission (demonstrating a diagnostic lag with a median lead time of 2.0 days). However, quantitative CEUS parameters obtained at baseline already displayed marked cortical hypoperfusion (reduced PI and TIC-AUC) and delayed microvascular transit (prolonged MTT), accompanied by significant elevations in systemic inflammatory, volume stress, and early renal injury markers (PCT, BNP, and sCys C). Extensive preclinical and clinical evidence confirms that systemic hemodynamic stability does not equate to microvascular adequacy (45), and CEUS-derived TIC parameters noninvasively quantify renal cortical microvascular perfusion (29, 30). Across animal models of ischemia-reperfusion and abdominal hypertension (19–23), as well as clinical cohorts of renal transplantation and AKI (24–26), reduced perfusion intensity and microbubble velocity consistently signify microvascular insufficiency. Specifically in septic populations, Watchorn et al. (27) and Harrois et al. (28) demonstrated prolonged MTT and reduced PI in septic shock patients with severe AKI, closely aligning with our observations.

Ultimately, the complex pathophysiological mechanisms underlying SA-AKI imply that single-dimension biomarkers or imaging modalities cannot resolve current diagnostic challenges (46). Integrating organ-specific microvascular perfusion metrics derived from quantitative CEUS with systemic clinical biomarkers into a multimodal predictive framework facilitates the identification of subclinical renal microvascular alterations, offering a potential diagnostic lead time of up to 48 h before standard clinical criteria are met. This may afford clinicians a critical window to optimize hemodynamic management and minimize potential nephrotoxic exposures.

To handle collinearity among quantitative CEUS parameters and mitigate overfitting in a small sample setting, we implemented Ridge logistic regression constrained by the conservative *λ*_1SE_ rule wth stratified 5-fold cross-validation (37, 38). Three hierarchical candidate models were constructed: Model A (CEUS-only), Model B (Clinical-only), and Model C (Combined Multimodal Model). The regularized regression coefficients of quantitative CEUS parameters displayed high stability within the model equation and demonstrated remarkable coefficient stability.

In model performance evaluation, compared with the high false-positive rate and potential over-intervention associated with the routine clinical model, the combined Model C achieved optimal diagnostic balance. Notably, its high negative predictive value underscores favorable clinical utility as a bedside rule-out tool. In calibration analysis, bootstrap resampling yielded calibration slopes exceeding 1.0 across all models. As highlighted by Riley et al. (47), applying penalization methods in small-sample settings can introduce hyperparameter estimation uncertainty, which frequently impacts calibration slopes. This probability shrinkage (under-dispersion) reflects the conservative *λ*_1SE_ penalization rule applied in a small-sample setting to prevent overfitting by shrinking regression coefficients toward zero. While this protective over-shrinkage compromises absolute probability calibration, Model C still maintains its discriminative power and risk-ranking capacity (bias-corrected AUC: 0.852), establishing it as an effective exploratory risk-stratification tool. Regarding overall prediction accuracy, Model C achieved a lower bias-corrected Brier score than the clinical model (0.194 vs. 0.225 for Model B), whereas the CEUS-only model (Model A) yielded a slightly lower Brier score (0.175), reflecting the trade-off of integrating a larger covariate set in a pilot cohort. Nevertheless, DCA confirmed that Model C provided a favorable standardized net benefit across a broad decision threshold spectrum (0.15–0.60).

To deconstruct independent predictors while mitigating small-sample bias and eliminating scaling effects, we conducted Z-score standardized (per 1-SD increase) Firth penalized likelihood regression. Perfusion intensity metrics—PI and TIC-AUC—emerged as strong independent protective factors. This mirrors the Ridge model formulations where perfusion intensity parameters consistently retained negative coefficients while injury and temporal metrics (sCys C, BNP, PCT, TTP, MTT) maintained positive coefficients, supporting previous literature (19–30) and confirming that reduced cortical microvascular perfusion is a core driver of SA-AKI (16–18).

Intriguingly, the temporal transit metric TTP did not reach statistical significance in univariable analysis (*P* = 0.095), but emerged as a significant independent predictor after adjusting for age and baseline sCys C. This unmasking of a suppression effect indicates that once confounding from baseline renal dysfunction (48–50) and age-related vascular stiffness (51) is removed, delayed microvascular inflow directly uncovers active microvascular vasospasm or capillary congestion. Conversely, the effect of MTT attenuated after multivariable adjustment, reflecting its collinearity with overall systemic illness severity. Echoing the findings of Shin et al. (50), these results emphasize that clinical interpretation of CEUS temporal parameters requires comprehensive evaluation within the patient's broader physiological context. Bedside quantitative CEUS captures a distinct pathophysiological dimension of renal cortical hypoperfusion independent of systemic inflammation (PCT) or cardiac/volume status (BNP). It serves not only as an independent predictor for inference but also provides reliable and incremental prognostic value for prediction in Model C.

Practically, Model C can be integrated into electronic health record (EHR) systems as a bedside risk calculator, providing an early warning window up to 48 h before standard clinical AKI diagnosis. At the optimal probability threshold of 0.417, the model offers clear clinical actionability: for high-risk patients (*P* ≥ 0.417), it triggers immediate bundled renal-protective strategies (e.g., avoiding nephrotoxic drugs and optimizing resuscitation); for low-risk patients (*P* < 0.417), its high negative predictive value (86.5%) helps rule out imminent SA-AKI, thereby preventing unnecessary over-resuscitation or over-intervention.

Several limitations of this study warrant consideration. First, given its single-center exploratory design and constrained cohort size, external validation in prospective, independent cohorts is essential, although internal validation using 1,000 bootstrap iterations demonstrated minimal model optimism. Furthermore, the limited sample size precluded subgroup analyses stratified by KDIGO severity stages (Stages 1–3). Finally, excluding patients with severe, refractory hemodynamic instability may have introduced selection bias, potentially limiting the generalizability of our findings to the most critically ill populations. Second, constrained by routine clinical sampling frequencies in real-world critical care (typically 1–2 times daily), our study could not capture the exact hour-level interval between CEUS examination and clinical AKI diagnosis. Third, RRI was not concurrently measured in this protocol, precluding a direct head-to-head comparison between macrovascular Doppler and quantitative microvascular CEUS parameters to evaluate their potential complementary diagnostic value. Future large-scale, multicenter trials encompassing the full spectrum of critically ill patients are required to confirm generalizability and support the development of a bedside clinical decision support tool.

## 5 Conclusions

Quantitative CEUS metrics (decreased PI and TIC-AUC, prolonged TTP) represent independent early predictive biomarkers for SA-AKI. Combining quantitative CEUS parameters with routine clinical biomarkers offers a promising strategy to identify high-risk patients with a potential lead time of up to 48 h prior to clinical diagnosis. Given the single-center, exploratory nature and limited sample size of this study, these early warning metrics and multimodal predictive models require external validation and recalibration in larger, multicenter prospective cohorts across the full spectrum of sepsis prior to clinical implementation.

## Data Availability

The raw data supporting the conclusions of this article will be made available by the authors, without undue reservation.
