# Supplementary material for: Early risk assessment model for sepsis-associated acute kidney injury combining quantitative contrast-enhanced ultrasound and biomarkers: a prospective pilot study
Source: Front Med (Lausanne). 2026 Oct 28;13:1991344. doi: 10.3389/fmed.2026.1991344 (PMC13751499; doi:10.3389/fmed.2026.1991344)
Supplement: Supplementary file 1 [file Table1.docx]

**Supplementary Table S1. Extended Baseline Characteristics and Clinical Outcomes**

| **Characteristic** | | **Overall (N = 63)** | **Non-SA-AKI (N = 38)** | **SA-AKI (N = 25)** | **P Value** |
| --- | --- | --- | --- | --- | --- |
| **Demographics & Physical Measurements** |  |  |  |  |  |
| Age, years | | 60.00  (50.00, 72.00) | 55.00  (52.00, 70.00) | 71.00  (48.00, 72.00) | 0.257 |
| Sex, Male, n (%) | | 52 (83%) | 29 (76%) | 23 (92%) | 0.176 |
| Ethnicity, Han, n (%) | | 59 (94%) | 35 (92%) | 24 (96%) | >0.999 |
| BMI, kg/m² | | 22.60(19.37, 26.67) | 20.76 (18.73, 24.61) | 24.22 (21.26, 26.81) | 0.343 |
| Occupation, n (%) | |  |  |  | 0.400 |
| $\quad$ Worker/Farmer | | 22 (35%) | 15 (39%) | 7 (28%) |  |
| $\quad$ Civil servant | | 4 (6.3%) | 3 (7.9%) | 1 (4.0%) |  |
| $\quad$ Company employee | | 12 (19%) | 7 (18%) | 5 (20%) |  |
| $\quad$ Self-employed | | 5 (7.9%) | 1 (2.6%) | 4 (16%) |  |
| $\quad$Retired/Unemployed/Other | | 20 (32%) | 12 (32%) | 8 (32%) |  |
| **Comorbidities & Lifestyle Factors** |  |  |  |  |  |
| Hypertension, n (%) | | 16 (25%) | 8 (21%) | 8 (32%) | 0.329 |
| **Diabetes mellitus, n (%)** | | **14 (22%)** | **5 (13%)** | **9 (36%)** | **0.033** |
| Liver injury/disease, n (%) | | 14 (22%) | 7 (18%) | 7 (28%) | 0.371 |
| Cardiovascular disease, n (%) | | 7 (11%) | 4 (11%) | 3 (12%) | 0.897 |
| History of malignancy, n (%) | | 13 (21%) | 5 (13%) | 8 (32%) | 0.071 |
| Prior abdominal surgery, n (%) | | 17 (27%) | 7 (18%) | 10 (40%) | 0.059 |
| Radiotherapy/Chemotherapy history, n (%) | | 7 (11%) | 4 (11%) | 3 (12%) | >0.999 |
| Pleural/Peritoneal effusion, n (%) | | 11 (17%) | 6 (16%) | 5 (20%) | 0.274 |
| Smoking history, n (%) | | 16 (25%) | 9 (24%) | 7 (28%) | 0.700 |
| Alcohol consumption, n (%) | | 16 (25%) | 9 (24%) | 7 (28%) | 0.700 |
| **Admission Source & Infection Characteristics** |  |  |  |  |  |
| Patient source, n (%) | |  |  |  | 0.773 |
| $\quad$ Emergency department | | 37 (59%) | 21 (55%) | 16 (64%) |  |
| $\quad$ Medical ward | | 11 (17%) | 7 (18%) | 4 (16%) |  |
| $\quad$ Surgical ward | | 15 (24%) | 10 (26%) | 5 (20%) |  |
| Primary site of infection, n (%) | |  |  |  | 0.139 |
| $\quad$ Pulmonary | | 43 (69%) | 24 (65%) | 19 (76%) |  |
| $\quad$ Intra-abdominal | | 16 (26%) | 12 (32%) | 4 (16%) |  |
| $\quad$ Skin and soft tissue | | 1 (1.6%) | 1 (2.7%) | 0 (0%) |  |
| $\quad$ Central nervous system | | 1 (1.6%) | 0 (0%) | 1 (4.0%) |  |
| $\quad$ Bloodstream | | 1 (1.6%) | 0 (0%) | 1 (4.0%) |  |
| **Clinical Severity Scores & Hemodynamics** |  |  |  |  |  |
| APACHE II score | | 20.00 (14.00, 28.00) | 20.00 (13.00, 28.00) | 21.00 (14.00, 28.00) | 0.994 |
| SOFA score | | 7.00 (5.00, 9.00) | 7.00 (5.00, 8.00) | 9.00 (5.00, 12.00) | 0.122 |
| GCS score | | 11.00 (6.00, 15.00) | 8.50 (4.00, 15.00) | 14.00 (9.00, 15.00) | 0.215 |
| Heart rate, beats/min | | 98.00 (86.00, 117.00) | 97.00 (81.00, 132.00) | 101.00 (95.00, 116.00) | 0.741 |
| Respiratory rate, breaths/min | | 16.00 (14.00, 20.00) | 16.00 (14.00, 20.00) | 18.00 (15.00, 22.00) | 0.364 |
| SpO₂, % | | 0.99 (0.97, 1.00) | 0.99 (0.97, 1.00) | 0.99 (0.97, 1.00) | 0.797 |
| Mean arterial pressure, mmHg | | 86.00 (80.67, 96.00) | 85.00 (79.00, 94.33) | 88.33 (84.00, 96.00) | 0.496 |
| **Central venous pressure, mmHg** | | **6.00 (5.00, 7.00)** | **6.00 (5.00, 6.00)** | **6.00 (6.00, 7.00)** | **0.024** |
| **Laboratory Biomarkers at Admission** |  |  |  |  |  |
| Urine output within 24h, mL | | 2500.00 (2020.00, 3148.00) | 2490.00 (2040.00, 3050.00) | 2600.00 (2020.00, 3310.00) | 0.584 |
| **Serum creatinine, μmol/L** | | **85.00 (66.00, 135.00)** | **69.50 (51.00, 85.00)** | **145.00 (129.00, 312.00)** | **<0.001** |
| **eGFR, mL/min/1.73m²** | | **78.62 (42.46, 99.48)** | **94.15 (81.94, 118.01)** | **38.08 (19.17, 45.69)** | **<0.001** |
| Blood urea nitrogen, mmol/L | | 10.60 (6.80, 19.10) | 11.20 (6.90, 20.00) | 9.30 (6.30, 17.70) | 0.762 |
| Uric acid, μmol/L | | 282.00 (141.00, 371.00) | 265.00 (166.00, 365.00) | 282.00 (141.00, 371.00) | 0.478 |
| **Serum cystatin C, mg/L** | | **1.21 (1.06, 1.87)** | **1.14 (1.00, 1.48)** | **1.84 (1.20, 2.37)** | **0.004** |
| **BNP, pg/mL** | | **1884.00 (786.00, 6541.00)** | **1252.00 (272.00, 3144.00)** | **5706.50 (1884.00, 8119.00)** | **0.001** |
| **Procalcitonin, ng/mL** | | **5.62 (0.88, 15.93)** | **2.45 (0.49, 12.78)** | **7.07 (3.78, 32.33)** | **0.012** |
| C-reactive protein, mg/L | | 140.00 (85.00, 212.00) | 132.00 (74.20, 187.00) | 187.00 (97.00, 235.00) | 0.135 |
| Interleukin-6, pg/mL | | 123.60 (38.00, 367.40) | 82.94 (36.88, 250.80) | 205.30 (47.06, 523.30) | 0.100 |
| **Treatments & In-Hospital Management** |  |  |  |  |  |
| Invasive mechanical ventilation, n (%) | | 47 (75%) | 28 (74%) | 19 (76%) | 0.836 |
| Duration of mechanical ventilation, hours | | 97.95 (0.00, 220.00) | 162.44 (0.00, 258.22) | 62.50 (0.50, 120.00) | 0.108 |
| Vasoactive/Vasodilator drug use, n (%) | | 20 (32%) | 9 (24%) | 11 (44%) | 0.090 |
| Analgesia and sedation, n (%) | | 25 (40%) | 16 (42%) | 9 (36%) | 0.628 |
| Diuretics, n (%) | | 14 (22%) | 9 (24%) | 5 (20%) | 0.731 |
| **Clinical Outcomes & Resource Utilization** |  |  |  |  |  |
| **14-day renal outcomes, n (%)** | |  |  |  | **<0.001** |
| $\quad$ No AKI | | 48 (76%) | 35 (92%) | 13 (52%) |  |
| $\quad$ Stage 1 AKI | | 6 (9.5%) | 2 (5.3%) | 4 (16%) |  |
| $\quad$ Stage 2 AKI | | 2 (3.2%) | 1 (2.6%) | 1 (4.0%) |  |
| $\quad$ Stage 3 AKI | | 7 (11%) | 0 (0%) | 7 (28%) |  |
| **28-day mortality, n (%)** | | **10 (16%)** | **3 (7.9%)** | **7 (28%)** | **0.042** |
| **ICU length of stay, days** | | **7.00 (4.00, 14.00)** | **8.50 (5.00, 19.00)** | **6.00 (2.00, 9.00)** | **0.009** |
| **Total hospitalization cost, RMB** | | **101,456 (51,225, 167,919)** | **58,675 (43,103, 127,410)** | **114,138 (74,475, 276,392)** | **0.002** |

**Note:** Continuous variables are presented as Median (Q1, Q3) and compared using the Wilcoxon rank-sum test. Categorical variables are presented as n (%) and compared using the Chi-square test or Fisher's exact test. **Abbreviations:** **APACHE II**, Acute Physiology and Chronic Health Evaluation II; **BNP**, Brain Natriuretic Peptide; **eGFR**, Estimated Glomerular Filtration Rate; **GCS**, Glasgow Coma Scale; **ICU**, Intensive Care Unit; **SOFA**, Sequential Organ Failure Assessment.
