# Supplementary material for: Early risk assessment model for sepsis-associated acute kidney injury combining quantitative contrast-enhanced ultrasound and biomarkers: a prospective pilot study
Source: Front Med (Lausanne). 2026 Oct 28;13:1991344. doi: 10.3389/fmed.2026.1991344 (PMC13751499; doi:10.3389/fmed.2026.1991344)
Supplement: Supplementary file 2 [file Table2.docx]

**Supplementary Table S2. Regularized regression coefficients (β) across candidate models (λ_1SE_ rule)**

| **Variable** | **Model A (CEUS-only)** | **Model B (Clinical-only)** | **Model C (Combined)** |
| --- | --- | --- | --- |
| **(Intercept)** | 1.7567 | -0.8048 | -0.8117 |
| **Demographic & Biomarkers** |  |  |  |
| Age (years) | — | 0.0013 | 0.0047 |
| BNP (pg/mL) | — | 0.0219 | 0.1197 |
| sCys C (mg/L) | — | 0.0571 | 0.3415 |
| PCT ($\mu$g/L) | — | 0.0177 | 0.1018 |
| **Quantitative CEUS Metrics** |  |  |  |
| PI, dB | -0.0737 | — | -0.0625 |
| TTP, s | 0.0073 | — | 0.0058 |
| MTT, s | 0.0123 | — | 0.0151 |
| TIC-AUC, a.u. | -0.0003 | — | -0.0003 |

**Note:** Regression coefficients (β weights) were generated using Ridge logistic regression constrained by the conservative 1-SE rule (λ_1SE_) under stratified 5-fold cross-validation. Em dashes (—) indicate variables not included in the respective model specification. *Abbreviations:* **BNP**, Brain Natriuretic Peptide; **CEUS**, Contrast-Enhanced Ultrasound; **MTT**, Mean Transit Time; **PCT**, Procalcitonin; **PI**, Peak Intensity; **sCys C**, serum cystatin C; **TIC-AUC**, Area Under the Time–Intensity Curve; **TTP**, Time to Peak.
